# Diabetes Self-Management Education and Support Culturally Tailored for African Americans: COVID-19-Related Factors Influencing Restart of the TX STRIDE Study

**DOI:** 10.1177/26350106211027956

**Published:** 2021-07-28

**Authors:** Mary A. Steinhardt, Sharon A. Brown, H. Matthew Lehrer, Susan K. Dubois, Jaylen I. Wright, Erum Z. Whyne, Lisa L. Sumlin, Louis Harrison, Jihun Woo

**Affiliations:** Department of Kinesiology and Health Education, College of Education, The University of Texas at Austin, Austin, Texas; School of Nursing, The University of Texas at Austin, Austin, Texas; Department of Psychiatry, School of Medicine, University of Pittsburgh, Pittsburgh, Pennsylvania; Department of Kinesiology and Health Education, College of Education, The University of Texas at Austin, Austin, Texas; Department of Internal Medicine, Dell Medical School, The University of Texas at Austin, Austin, Texas; Department of Kinesiology and Health Education, College of Education, The University of Texas at Austin, Austin, Texas; Department of Kinesiology and Health Education, College of Education, The University of Texas at Austin, Austin, Texas; School of Nursing, The University of Texas at Austin, Austin, Texas; Department of Curriculum & Instruction, College of Education, The University of Texas at Austin, Austin, Texas; Department of Kinesiology and Health Education, College of Education, The University of Texas at Austin, Austin, Texas

## Abstract

**Purpose:**

The purpose of this substudy was to determine the most acceptable way to restart the Texas Strength Through Resilience in Diabetes Education (TX STRIDE) study safely using remote technologies. Following the emergence of COVID-19, all in-person TX STRIDE intervention and data collection sessions were paused.

**Methods:**

Qualitative descriptive methods using telephone interviews were conducted during the research pause. A structured interview guide was developed to facilitate data collection and coding. Forty-seven of 59 Cohort 1 participants were interviewed (mean age = 60.7 years; 79% female; mean time diagnosed with type 2 diabetes = 11 years).

**Results:**

Data categories and subcategories were generated from the interview responses and included: personal experiences with COVID-19, effects of COVID-19 on diabetes self-management, psychosocial and financial effects of COVID-19, and recommendations for program restart. Although some participants lacked technological knowledge, they expressed eagerness to learn how to use remote meeting platforms to resume intervention and at-home data-collection sessions. Six months after the in-person intervention was paused, TX STRIDE restarted remotely with data collection and class sessions held via Zoom. A majority of participants (72.9%) transitioned to the virtual platform restart.

**Conclusions:**

Qualitative findings guided the appropriate implementation of technology for the study, which facilitated a successful restart. High retention of participants through the study transition provides evidence that participants are invested in learning how to manage their diabetes despite the challenges and distractions imposed by COVID-19.

Type 2 diabetes (T2D) is the third leading cause of years lived with disability in the United States,^1^ costing $327 billion annually ($1 in $7 health care dollars) and accounting for the highest health care expenditures among all adult disease categories.^2^ By 2050, T2D is projected to affect 33% of the US population.^3^ African Americans (AAs) bear a disproportionate burden of the T2D epidemic, with double the prevalence^4^ and a 64% higher incidence in comparison with non-Hispanic whites.^5^ Among AAs, T2D-associated stress (diabetes distress) is often compounded by general life stress, which exacerbates poor glucose control.^6^ Effective interventions, culturally tailored for AAs with T2D, are urgently needed to address this ongoing and growing health disparity and to adapt to additional demands of the novel coronavirus disease 2019 (COVID-19) pandemic.

Texas Strength Through Resilience in Diabetes Education (TX STRIDE), a National Institutes of Health-funded diabetes self-management randomized controlled clinical trial, addresses this major health disparity in AAs, T2D, and its devastating consequences. Psychosocial resilience, the ability to bounce back in the face of adversity,^7^ may improve individuals’ capacity to successfully manage the daily challenges of a diabetes diagnosis and related complex self-management responsibilities. Individuals who utilize psychosocial resilience resources (eg, positive affect, spiritual coping, emotional regulation, social support, self-efficacy) are better able to reduce T2D-associated stress.^8,9^ Research has documented an association between the use of psychosocial resilience resources and lower mortality risk in individuals diagnosed with T2D.^10^ Teaching resilience skills in diabetes self-management education and support (DSMES) programs in the AA community may be particularly valuable because AAs value resilience as a cultural skill learned through years of coping with discrimination and racism.^11,12^

TX STRIDE is the first longitudinal study to examine whether a resilience-based diabetes self-management education and support (RB-DSMES) program (vs a traditional DSMES program) improves psychosocial resilience resources and whether enhanced resilience resources improve T2D physical and mental health outcomes. Study participants are recruited through AA churches and the 10-month TX STRIDE intervention program (ie, 8 weekly class sessions followed by 8 biweekly support group sessions followed by 2 bimonthly booster sessions) is conducted at those churches. Cohort 1 (N = 59) began in January 2020 with baseline data collection followed by in-person small group educational classes at 4 churches: 2 in the RB-DSMES experimental arm and 2 in the DSMES control arm. An overview of the RB-DSMES and DSMES curriculum is provided elsewhere.^9,13^

Following the emergence of COVID-19, all research on campus was paused in March 2020, at which time the Cohort 1 experimental arm (n = 14) had completed 4 class sessions and the control arm (n = 45) had completed 6 class sessions. Attendance for all 4 groups was strong at just over 90%. As of March 2021, TX STRIDE remains paused for all in-person activities.

The ongoing COVID-19 pandemic and its associated consequences resulted in feelings of fear, stress, and anxiety among the population. AAs with T2D are particularly vulnerable to serious negative health outcomes of COVID-19 because diabetes is a major risk factor for rapid progression of COVID-19.^14-17^ Additionally, AAs may have inadequate access to health care and increased exposure to COVID-19 because they are more likely to be employed as essential workers compared to non-Hispanic whites.^14^ Individuals diagnosed with T2D and no other comorbidities appear more susceptible to rapid deterioration from COVID-19 even after controlling for age.^15^ Systematic reviews have identified COVID-19 patients with T2D, compared to patients without T2D, to be at 3 times higher risk of intensive care unit admission, invasive ventilation, and death.^16-18^ Evidence is also emerging that obesity and higher glycated hemoglobin (A1C) are linked to worse COVID-19 outcomes (eg, hospitalization, in-hospital death).^19^

In addition to the direct risks of COVID-19 to individuals with T2D, stay-at-home orders to curb the spread of the pandemic also contribute to worse diabetes outcomes due to higher levels of stress and reduced physical activity, healthy dietary intake, and routine medical care. Comprehensive diabetes care has taken a secondary role to concerns regarding the spread of COVID-19.^20,21^ It was crucial to resume TX STRIDE classes as soon as possible after the research pause mandate due to the pandemic because addressing disparities in T2D and helping AAs maintain glycemic control could help prevent, or at least reduce, the grave consequences of COVID-19 and lessen existing health disparities.^19^

Therefore, a descriptive qualitative study involving telephone interviews with participants enrolled in TX STRIDE when the study was paused due to COVID-19 (Cohort 1, N = 59) was conducted to: (1) determine the most acceptable way to safely restart the TX STRIDE program (ie, evaluate virtual options and preferences), (2) inform any necessary changes to the curriculum content related to COVID-19 and its relationship with T2D, and (3) identify any new health or logistical challenges to be considered during the remote restart of the TX STRIDE program.

## Methods

### Research Design

The present study used a basic qualitative descriptive research design. The purpose of this design is to provide a comprehensive summary of individuals’ experience using their own words that can be easily understood. Data are organized in a logical manner and presented as a descriptive summary of straightforward answers to questions that are of practical relevance to restarting a DSMES intervention during the ongoing COVID-19 pandemic.^22^

### Procedures

Individual interviews were scheduled by 3 TX STRIDE research team members who were familiar with study participants and had previous training in and experience with conducting qualitative interviews. Participants were contacted via telephone because in-person data collection was paused due to COVID-19. All interviews occurred during May and June of 2020 and lasted approximately 30 to 45 minutes each. Participants were read a consent statement and provided verbal informed consent prior to the interview. All interviews were audio recorded and did not contain any personally identifiable information, including names. After completion of the interview, each interviewee was emailed a $30 gift card to compensate for the time involved in participating. All procedures were approved by the Institutional Review Board of The University of Texas at Austin.

A structured interview guide was developed consisting of 11 open-ended questions (see Table 1) with probes designed to guide the interviews to generate specific, actionable suggestions for restarting TX STRIDE safely during the ongoing COVID-19 pandemic. Participants were given the opportunity to raise other related topics if desired. The interviewers met twice with the study investigators to become familiar with study procedures and refine the interview questions.

**Table 1:** Interview Questions for Participants

No.	Interview question
1	Have you experienced any COVID-19 symptoms (eg, fever, tiredness, dry cough, aches and pains, sore throat) or been told you have or likely have COVID-19?
2	If you have not had COVID-19, how concerned are you that you will get it?
3	Have any aspects of your diabetes treatment and self-management been affected by the COVID-19 pandemic and mandated government guidelines? If so, in what ways?
4	What barriers related to the COVID-19 pandemic have prevented you and others you know from adopting or sustaining diabetes self-care activities?
5	How are you currently getting groceries for your home during this pandemic?
6	Where have you received most of your information regarding COVID-19 (eg, TV, Internet, social media, family, friends, newspapers, church)? What information have you received about COVID-19 and diabetes?
7	How has the COVID-19 pandemic affected you and your family financially?
8	Are you able to participate in Sunday church services and other activities remotely? If so, what platform (eg, Zoom, YouTube) do you use to participate?
9	What are your thoughts on using some type of technology as part of the diabetes self-management program? Do you have access to any of these?
10	What recommendations do you have that would help us restart the diabetes program when safe to do so?
11	We know the COVID-19 pandemic may be affecting your life or community in important ways we have not thought to ask about. Is there anything additional that you would like to share?

### Recruitment and Sample

Participants from Cohort 1 of TX STRIDE were contacted by text messages and telephone calls to request their voluntary participation. Forty-seven of the 59 Cohort 1 participants agreed to the interview, for a participation rate of 80%. Reasons for decline included not being interested in this additional TX STRIDE data-collection opportunity (n = 2), death (n = 1), dropped out prior to COVID-19 (n = 1), and lack of a response to the initial participant contact via text message or telephone call (n = 8).

### Data Analysis

Data analysis followed established procedures for analyzing qualitative descriptive data.^22,23^ Audio recordings of the interviews were transcribed by professional research transcriptionists. The transcriptions were reviewed by the authors for accuracy and then coded, supplemented with the audiotapes. Data were highly structured, consistent with the 11 questions asked of participants and thus easily coded and synthesized. Three of the authors reviewed all transcript data independently and summarized their initial impressions. All coding discrepancies were discussed by the authors until consensus was achieved. Descriptive statistics of demographic data were summarized using SPSS Version 26.0 (Armonk, NY).

## Results

### Participants

Interviews were completed by 47 participants. The majority were female (n = 37, 78.7%) and married (n = 28, 59.6%). Approximately one quarter of the sample had a college degree (n = 12, 25.5%). Participants ranged in age from 41 to 85 years (mean = 60.7, SD = 8.8) and had been diagnosed with T2D from 0.5 to 32 years (mean = 11.4, SD = 9.0). See Table 2 for additional participant information.

**Table 2:** Demographic Characteristics of Study Sample (N = 47)

Characteristics	Mean (SD) or %	No.
Age	60.68 (8.80)	47
Sex (% female)	78.7%	37
Diabetes diagnosis length (y)	11.35 (9.01)	46
Marital status		
Never married	6.4%	3
Married	59.6%	28
Separated/divorced	29.8%	14
Widowed	4.3%	2
Highest education level attained		
High school graduate/GED	27.7%	13
Some college/technical school	46.8%	22
College graduate/bachelor’s degree	19.1%	9
Graduate degree	6.4%	3
Employment status		
Full-time	44.7%	21
Part-time	4.3%	2
Unemployed/laid off (looking for work)	2.1%	1
Unemployed/laid off (not looking for work)	6.4%	3
Retired	42.6%	20
Household income		
≤$19 999	10.6%	5
$20 000-$39 999	25.5%	12
$40 000-$59 999	29.8%	14
$60 000-$79 999	19.1%	9
$80 000-$99 999	6.4%	3
≥$100 000	6.4%	3
Previously attended diabetes classes (% yes)	44.0%	16
Ever used tobacco products (% yes)	72.3%	34
Currently using tobacco products (% yes)	6.4%	3
Days per week with at least 30 min spent in physical activity	2.53 (1.82)	47
Blood pressure medication (% yes)	89.4%	42
Cholesterol medication (% yes)	57.4%	27
Diabetes-related medication use (% yes)		
Oral meds and/or noninsulin injectable only	55.3%	26
Insulin only	12.8%	6
Both	23.4%	11
None	8.5%	4

### Qualitative Categories

Created categories captured participants’ views for restarting the TX STRIDE program virtually as well as health and logistical challenges resulting from the pandemic that would need to be considered upon program restart. The final 5 categories are discussed in the following, and deidentified quotes are inserted in the narrative to support and/or clarify perspectives from the individual telephone interviews.

#### Category 1: Personal experiences with COVID-19

##### Diagnosis and symptoms

No participant was diagnosed with COVID-19 at the time of the telephone interviews or reported symptoms unique to COVID-19. Participants were aware of potential COVID-19 transmission from family members, particularly from family members working outside the home. Participants expressed concern regarding their risk for COVID-19 and described the daily strategies they employed to mitigate that risk, such as wearing masks, washing hands, and having family members wash their clothes and shower upon returning home from work.

##### Sources of COVID-19 information

The majority of participants received information about the pandemic from television, social media, church, and friends/family members. Primary trusted sources of news included health care professionals interviewed on local and national TV programs (particularly experts in viral diseases), representatives of the Centers for Disease Control and Prevention, church or work websites, or family members in the medical field. Several participants commented that although the news was helpful, sometimes it was confusing, contradictory, or not factual. Others avoided watching the news for excessive periods of time because it was disturbing and/or anxiety-provoking. For example, one participant commented: “I start feeling my anxiety kick in when I be getting too much information, so I turn it [off].” Although several participants gave voice to the deeply rooted and justified widespread mistrust that many AAs have with regard to health care and clinical research,^24^ participants also relied on experts from these fields for information on COVID-19.

##### Participant perspectives of COVID-19

All participants perceived the virus to be a serious threat, and the vast majority felt that living with T2D put them at increased risk for COVID-19 and a worse prognosis if infected. To the extent possible, participants followed suggested preventive measures (eg, washing hands, staying at home). One participant had a sign on her front door that read: “Nobody can enter my house without a mask. That’s including kids.” Another participant recalled an experience trying to protect himself while shopping for groceries:

And sometimes, like going down the aisle, with somebody that’s not wearing a mask, I speak for me, I’m so concerned, I’ll turn my basket around and go the other way. Because that way, I guess I’m not fearful, but I’m careful and cautious.

Although participants took practical precautions, they often balanced them with acknowledgment of their spiritual beliefs. For example, one participant said: “If it’s our time to go, it’s our time.” Another participant commented: “If I were to get it, then it would be because it’s His will, and whatever He brings me to, He always takes me through.”

With respect to employment, some participants were laid off from work or furloughed. Some participants transitioned to working from home, whereas others continued to attend their regular work site because many were designated as essential workers. Many family members of essential workers expressed concerns regarding the public’s behavior:

My husband works for a grocery store. Well, he just said that people are not really serious about what’s going on because some people are not wearing masks, getting too close to them, but he wears his mask. It’s kind of frightening, knowing that he’s out there.

#### Category 2: Effects of COVID-19 on diabetes self-management

##### Dietary behavior

COVID-19 impacted the shopping patterns of many participants. Long lines at the grocery store and safety issues related to shoppers not following recommended protections (eg, social distancing) created concerns when shopping for groceries. Participants adjusted their shopping routines to enhance safety and avoid waiting in long lines. Many participants shopped early in the morning or during the reserved hour for senior citizens. Others experimented with ordering groceries online and picking them up curbside or having them delivered. Notably, many participants described a strong, and sometimes large, social network of family (eg, children, grandchildren) and friends or neighbors who shopped for them. Others preferred to grocery shop themselves, often creating a list and going once a week or once every other week. One participant commented: “But you got no choice. If you want your groceries you know what I’m saying . . . I really had to develop my patience.”

Many participants reported that being confined to their homes all day resulted in them snacking more or grazing all day. For example, one participant noted that working from home made it easy to develop “a habit of walking to the refrigerator and back to the computer to work.” Others attributed a slight weight gain to increased snacking and a lack of physical activity due to being “stuck” in one place.

##### Participating in physical activity

Several participants reported that the fitness tracker watch they received as part of the TX STRIDE program helped them stay motivated to reach their daily step targets. For example, one participant commented, “It’s on my arm. I only take it off when I need to charge it.” Another said, “Yeah. I keep that on. It’s keeping me in line, I think.” Others leaned on family members and friends for support, participated in exercise classes virtually, or had jobs that required a lot of walking. Still others did their best to boost their activity by walking around the house or down to the mailbox, as expressed by the following participant: “I’m getting up to my 10 000 steps per day. I go out when it’s fair and walk up and down in front of my house and then around in my house all day.”

Several participants expressed challenges with physical activity and voiced that during the pandemic they were not walking as much as they used to. One participant said, “I’m just not motivated to do it. Once I’ve finished my work day around here, sitting around all day and behind the computer, I don’t want to do anything.” Others stated that they missed the structure of the weekly TX STRIDE class sessions: “I’m not quite as conscientious as I was when I was in the class. The structure and timing. It kept me a lot more conscientious.”

##### Medication adherence and self-monitoring of blood glucose

TX STRIDE participants were provided a glucometer and testing supplies. Any participant without insurance or a health care provider was referred to a local community health center to receive diabetes care from the endocrinologist, a team member of TX STRIDE. A majority of participants reported getting their prescriptions refilled, adhering to their medication regime, and regularly testing their blood glucose levels. Several participants commented that decreased physical activity levels and increased snacking during the pandemic were reflected by an increase in blood glucose readings. One participant’s A1C had gone back up “since all this mess . . . they changed my insulin.” A few participants were low on medication supplies or concerned about ongoing medical care issues.

##### Routine medical care appointments

Many participants reported meeting with their health care provider over the telephone during the pandemic. Others went in person to the physician’s office or clinical lab for blood work or for their regular appointments. Most participants felt safe, as reflected in this comment: “Yes. I’ve gone in for my annual checkup with my doctor. . . . I felt it was safe . . . because they’ve used the distancing measures and I’ve used my mask and my gloves.” One participant was opposed to having her appointment virtually, stating: “I’m not computer savvy and you need to be computer savvy. . . . You know, I can’t see you. You can’t examine me on the screen, I’m sorry.”

#### Category 3: Psychosocial effects of COVID-19

##### Feelings of vulnerability

The impact of COVID-19 and associated vulnerability resulted in feelings of anxiety among participants, especially given the increased risk of this vulnerable population to COVID-19. For example, one participant commented:

It really was frustrating, because say you watched the news . . . it’s affecting mostly African American[s] and you know you’re one. . . . You’re like, “Oh God, I don’t even want to go outside, but I have to go.” And it was, at first, it was really intense for me.

The ongoing stress of the pandemic on participants’ mental health affected their diabetes self-management behaviors. One participant commented: “I’m eating something that I know I’m not supposed to be eating, but it’s just that with my anxiety.” Another said: “I try not to be out and about that much, but I’m about ready to go stir crazy in this house though.” Helpful strategies included having a positive mindset and trying to make the best of a difficult situation, for example, focusing on projects around the house, cleaning out a closet, or taking a short walk. Creating a routine was suggested as important for maintaining one’s health amid the adversity caused by the pandemic:

Even if you enter a dilemma situation like this coronavirus thing that can change your mentality and everything else. We got to keep a schedule . . . if you deviate from the routine, just get back on it as soon as possible, and that will keep you focused.

This participant’s comment was reflective of the resilience strategies emphasized during the TX STRIDE in-person class sessions prior to the pandemic.

##### Ability to participate in church services

The 4 churches in Cohort 1 found ways to connect with and support their church members by successfully converting to virtual services. Church members were provided instructions for using these platforms and were highly motivated to learn how to use them to maintain a connection with their church. The most commonly used platforms included Zoom, Facebook Live, YouTube Live, and conference calls. Although socially distanced in-person services resumed at some churches in May 2020, many participants continued to prefer attending church gatherings virtually, as reflected in this comment: “A lot of us feel we go to church and we protected, we’re not going to get it, and that’s not a true fact, it don’t discriminate.”

##### Social support from family and friends

The support of loved ones was critical as participants tried to adapt to the ongoing stress of COVID-19. One participant commented that her husband encouraged physical activity, saying, “Let’s get up and go walk.” Another participant said that her family is “making sure I’m taking my insulin.” Still another, reflecting on the support provided by her daughter and grandchildren that lived with her, said: “If I find myself where I can’t do something or need help with something, yeah, they’re definitely there to support that.”

Support from friends and church members also helped buffer the adverse effects of the pandemic on participants’ health. One participant commented: “Yes, I have some church friends that I always go to.” Another participant said that in the beginning she “lost the courage to walk and watch what I eat.” It was church friends that helped her bounce back:

I bounced back and then I was calling others that was in the [TX STRIDE] class and make sure they was doing it. . . . I said, “Okay, I’m going to keep them on track,” and so that put me back in track when I would call them and ask them, “Are you keeping up with your exercise?” And they say, “We’re doing it now.”

#### Category 4: Financial effects of COVID-19

Several participants had successfully transitioned to working from home and were not impacted financially, as evidenced by this comment: “I have not lost any pay since this has started so my finances hasn’t been affected. And that goes from my entire family. Like I said, I’m blessed.” Alternatively, the adverse financial impact of COVID-19 on many participants was evident and contributed to increased stress levels. One participant said: “My husband’s [job] was not essential. So, his job went into shelter-in-place mode back in March . . . he hasn’t had any income.” Another commented:

I was working and I got laid off, so yes, it’s very stressful. . . . And even before they say anything about things with money or whatever, you’d be like, “Oh lord, what am I [to] do?” When they came to you, it was like, I was stunned.

Many participants were not able to work from home, resulting in a greater chance of exposure to the virus while simultaneously feeling like they couldn’t afford to miss a day of work. Family members across generations often lived in 1 household, providing social support and financial savings yet also making it more challenging to adhere to COVID-19 prevention protocols. Although some participants experienced challenging financial situations, family and community members worked together to support each other and obtain needed resources, as evidenced by this comment: “My husband, because he always go to the store and get canned goods just stacking them. . . . Say somebody be low on this, I got it. The bag is on the porch, come by.” Another participant commented:

I’ll help a family member . . . we had to step up and help them [grandchildren] out. We’ve been going to the schools and they get their lunches. We’ll pull up to the school and they’re helping them with snacks and whatever. . . . That’s a support system I would say that we been tapping on since the COVID crisis.

Government stimulus checks, unemployment benefits, and other resources as a result of lost employment provided some relief. Although completing and submitting the paperwork was challenging, one participant commented: “My son. He got laid off his job. My grandson got laid off his job . . . we’re trying to help him file unemployment. [He] couldn’t get through his claims. I was able to help.” Support that was provided was needed and appreciated, as reflected by this participant comment: “The stimulus check came through. That was perfect, right on time, because I was behind.”

#### Category 5: Recommendations for TX STRIDE program restart

##### Face-to-face versus remote options

Participants missed the education and support provided by the diabetes program and were eager to resume the classes. For example, one participant said:

I really miss the class. I enjoyed it. When you go and listen to everybody’s story, at least you know you’re not alone. . . . Hoping we can pick it back up soon. . . . For me, it was very educational. You know, I learned a lot.

Participants proposed options for a safe restart, including a few comments from participants willing to resume in-person classes. For example, one participant stated: “As long as everybody wear their mask. I’ll be comfortable with it like that.” However, given the vulnerability of participants to COVID-19 and their increasing agency in attending virtual church services and other church activities, the general consensus was to restart the classes remotely: “Do it on Zoom. I would feel more safe.” This comment was aligned with the decision made by several churches, who were also trying to determine how best to support their congregations during the pandemic. Holding Bible study and other church meetings via Zoom emerged as a commonly used platform. One participant’s comment reflects this perspective:

Only thing I can say is to do the Zoom deal. Because if you want to talk to people and you want to be able to see them, that’s a great way of getting in touch with people. . . . So yeah, that would be the closest to being in the same room with somebody, is the Zoom.

##### Access and acclimation to technology

Although not all participants had access to computers and smartphones, with help from family and church members, participants had been motivated to learn how to access church services. Furthermore, participants had experienced success in joining church events, which contributed to their confidence and motivation to explore remote options for restarting the diabetes program and for staying engaged. For example, one participant said, “My son . . . he’s computer savvy. He would set it up for me and I’d do my lesson.” Another stated: “I don’t have an iPad, and my phone was not one of those iPhones my granddaughter said. So, I can do the calling in and listen or whatever.” Participants conveyed a genuine willingness to stay connected and were open and honest regarding their current experiences using technology and obtaining needed information. One participant stated:

I got Internet here. My son and granddaughter comes a lot, and I keep it for them. But ain’t a thing I know about it and can do with it . . . me and the computer don’t do too good. Yeah. I’ve heard of Zoom. We do our BSF, Bible Study Fellowship, I’m a member of that, and we did it on Zoom too. But I was never able to get my phone to work and get it.

It was evident that some participants attending church meetings with access to both a smartphone, tablet, and/or computer had tried various options to maximize their experience using Zoom. And they seemed very willing to help other church members. For example, one participant said:

Yeah, I use my iPad because I can see like 9 people at one time . . . including yourself would be nine. On the [smartphone] you can only see maybe yourself and 1 other person. Or in like a pack of 4. So, it’s bigger, I get to see more on my iPad.

## Discussion

The culturally tailored RB-DSMES intervention, TX STRIDE, is intended to address a major health disparity among AAs, T2D, and its devastating consequences. Following the emergence of COVID-19, TX STRIDE was paused for in-person activities. Findings from this qualitative study guided the appropriate implementation of technology for TX STRIDE and facilitated a successful remote restart of the intervention program and data-collection activities.

During interviews, many participants reported listening to medical professionals’ advice while also expressing skepticism about the health care system. Historically, AAs have been disproportionately affected by public health crises compared to other racial/ethnic groups, and the current COVID-19 pandemic is no exception.^25^ Although AAs constitute 13% of the US population, they have accounted for 21% of deaths from COVID-19.^25^ Hartmann-Boyce et al^19(p1695)^ caution that “there is a high potential for COVID-19 to exacerbate existing health disparities, and research and practice guidelines need to take this into account.” Culturally competent interventions must include honest conversations regarding racial discrimination, cultural insensitivity, distrust of medical providers based on historical mistreatments, and barriers to care.^19^

When the TX STRIDE program restarted remotely in August 2020, instructors allowed participants to voice any feelings of mistrust and negative experiences in a safe and supportive environment. Instructors also allowed time to discuss information regarding COVID-19 vaccines, best practices for being safe, and the susceptibility to COVID-19 among people with T2D. Instructors shared their experiences receiving the COVID vaccine or plans to receive the vaccine, and the team endocrinologist attended class to listen, answer participant questions, and provide information on how to receive the vaccine. This information was also posted on the TX STRIDE Facebook page for each church and provided via email and text messages. After the program restart, 2 TX STRIDE participants tested positive for COVID-19 and reported serious symptoms. In classes, they shared their experiences of how the diabetes classes had enabled better diabetes self-management during this extremely stressful time.

Disruptions caused by COVID-19 contributed to changes in participants’ diabetes self-management behaviors and psychological well-being. Some participants mentioned that being stuck at home all day increased their access to food and, subsequently, frequent snacking and also restricted physical activity, leading to weight gain. Heightened stress during the pandemic was reported by study participants, and stress and emotional dysregulation are known to affect glycemic control.^19,21^ At the same time, participants reported that the social support from group members, resilience resources, and their fitness tracker were beneficial in adapting to the stress of the pandemic. TX STRIDE encourages walking as a safe form of exercise for its population. In response to social-distancing guidelines, the program developed physical activity cards and videos that detail simple exercises participants can do with limited space and equipment in their home. Mindful eating activities to support participants’ healthy relationship with food and address the negative impact of weight stigma and weight cycling were also developed.^26^

The TX STRIDE program emphasizes the building of resilience resources to help individuals adhere to healthy lifestyle changes. Despite the resilience that AAs have found useful in adapting to stressful situations, Novacek et al^24^ argue that extra attention be focused on the unique behavioral health needs of AAs during the COVID-19 pandemic, including: noticing changes in routine medical care, such as insurance coverage and prescription refills; addressing mental health issues; monitoring access to healthy foods and self-monitoring of blood glucose; and encouraging unique ways of engaging in physical activity.^19^ Additionally, attention to the financial effects of COVID-19 must be addressed because participants may be forced to ration medications or food supplies to pay costs-of-living expenses such as utility bills or rent.

Of all the resilience resources emphasized by study participants as being important for their well-being, 2 of the most prominent resources during the pandemic included spiritual coping and the social support received from family, friends, neighbors, and the church community. This finding is consistent with resilience resources that contributed to the well-being of AAs following Hurricane Katrina^27^ and quality-of-life indicators in AAs who tend to reside in high-risk urban communities.^28^ When TX STRIDE restarted remotely, instructors allowed time for opening and/or closing class sessions with prayer and encouraged participants to allow family members to join for remote sessions if desired.

TX STRIDE, like most health care services, had to determine how to reach its participants virtually. Telehealth, the use of electronic information and telecommunications services to deliver health care services including self-management education, has benefits including lower cost to provide services and the ability reach more people, particularly those that may face barriers to attending in-person services. Barriers to in-person attendance, often due to travel or financial constraints, are greater for minority, lower income, and senior populations. Yet these same populations also face barriers to telehealth, known as the digital divide, such as inadequate access to technology, unreliable Internet coverage, and low digital literacy.^29^ Because TX STRIDE serves these populations, there was concern about losing participants due to the digital divide. However, the interviews demonstrated that most participants had access to devices (ie, smartphones, tablets, or computers) and the Internet, either directly or through family members. Although many participants identified themselves as not being “technically savvy,” they were eager to learn how to use technology to continue with TX STRIDE and access other services. Many participants had also gained agency through learning how to virtually attend churches services and other church activities that were now virtual.

Zoom emerged as the platform of choice because churches were most commonly using this platform for Bible studies and other church services and meetings. A protocol was developed, and dedicated staff helped participants learn how to set up and use Zoom, often relying on the support of participants’ family members to help connect participants via Zoom. In some cases, the family member was necessary only at the initial setup, and in other cases, the family member was needed more frequently. Participants also expressed a willingness to participate in at-home data collection sessions with help from the TX STRIDE staff via Zoom or telephone. To further support remote classes, all curriculum materials were printed and mailed to participants before the restart of the program.

## TX STRIDE Restart

In August 2020, six months after all in-person intervention sessions and data-collection sessions were paused due to COVID-19, TX STRIDE restarted remotely, and baseline data were collected again (ie, baseline restart). This data collection was performed remotely with a research staff member assisting via Zoom or telephone. Following data collection, in September 2020, TX STRIDE class sessions began on Zoom. Of the original 59 participants recruited, 43 (72.9%) participated in the restart. All 43 participants are currently still participating in the program and have completed the 8 weekly class sessions and 8 biweekly support group sessions. Originally, 37 participants joined the Zoom classes with video, and 6 joined by calling over the telephone; but with a few weeks of experience, 4 of the 6 call-in participants moved to video. For a variety of reasons, 16 participants who attended the original in-person program did not rejoin when the remote program restarted. Reasons included: non-COVID-related death (n = 1), job conflict that began prior to the pandemic (n = 1), technology-related issues (n = 2), medical- and technology-related issues (n = 4), could not make the time commitment (n = 3), and other personal reasons (n = 5). Nevertheless, it is remarkable that more than 70% of this older population, with average household incomes of less than $60 000 per year, were able to transition to the virtual platform for the TX STRIDE restart. This investment in the use of technology has other potential health benefits, including staying connected to friends and family and engaging in virtual visits with their health care providers. The importance of these visits has been magnified during the COVID-19 pandemic.

Results and interpretations of the present study should be considered in light of several limitations. First, although 80% of participants in Cohort 1 participated in telephone interviews, their individual perceptions may not represent all Cohort 1 participants or future participants. In addition, selection bias may have existed because those who participated may have been more eager to discuss their experiences during the COVID-19 pandemic and/or more eager to resume the program virtually.

## Implications

Findings identified common perspectives and suggestions that together guided the appropriate implementation of technology in the TX STRIDE sample and facilitated a successful remote restart. Furthermore, and perhaps most importantly, this qualitative study generated strong evidence that participants were fully engaged in learning how to effectively manage their diabetes despite the challenges and distractions imposed by COVID-19. Designing culturally tailored interventions specifically for AAs that build on their inherent motivation and resilience are more likely to succeed in addressing existing health disparities related to T2D and COVID-19.
